# Streptothrix Possible Cause of a Banti’s Disease

**Published:** 1914-08

**Authors:** 


**Streptothrix Possible Cause of a Banti's Disease.** Gibson, Proceedings of Royal Soc. of Med., claims to have demonstrated mycelia in the spleen by Wheal and Chorm's method, but has not succeeded in making cultures.

